# Experimental Study on Thermal Conductivity of Self-Compacting Concrete with Recycled Aggregate

**DOI:** 10.3390/ma8074457

**Published:** 2015-07-20

**Authors:** María Fenollera, José Luis Míguez, Itziar Goicoechea, Jaime Lorenzo

**Affiliations:** 1 Department of Design in Engineering, Industrial Engineering School, University of Vigo, Vigo 36310, Spain; E-Mail: igoicoechea@uvigo.es; 2 Department of Mechanical Engineering, Machines and Thermal Engines and Fluids, Industrial Engineering School, University of Vigo, Vigo 36310, Spain; E-Mail: jmiguez@uvigo.es; 3 Department of Materials Engineering, Applied Mechanics and Construction, Industrial Engineering School, University of Vigo, Vigo 36310, Spain; E-Mail: jaimelorenzo@uvigo.es

**Keywords:** self-compacting concrete, recycled aggregate, thermal conductivity, infrared thermography

## Abstract

The research focuses on the use of recycled aggregate (RA), from waste pieces generated during production in precast plants for self-compacting concrete (SCC) manufactured with a double sustainable goal: recycle manufacturing waste (consumption) and improvement of the thermal properties of the manufactured product (energy efficiency). For this purpose, a mechanical study to ensure technical feasibility of the concrete obtained has been conducted, as well as a thermal analysis of recycled SCC specimens of 50 N/mm^2^ resistance, with different RA doses (0%, 20%, 50% and 100%). The main parameters that characterize a SCC in both states, fresh (slump-flow) and hard (compressive strength), have been tested; also, a qualitative analysis of the thermal conductivity using infrared thermography (IRT) and quantitative analysis with heat flow meter at three temperatures 20 °C, 25 °C and 30 °C have been performed. The results suggest the existence of two different thermal behaviors: concretes with 0% and 20% of RA, and on the other hand concretes with 50% and 100% of RA. It has also demonstrated the validity of the IRT as sampling technique in estimating the thermal behavior of materials having reduced range of variation in parameters.

## 1. Introduction

Although concrete is the most commonly used construction material anywhere in the world, it is not considered sustainable because of its destructive nature in the consumption of natural resources and the environmental impact after its use. Approximately 65% of the volume of concrete is aggregate; therefore discovering suitable substitutes for natural aggregates is a priority. By this way, for the construction industry, reusing concrete waste may be an attractive option for the good practice of producing high quality eco-concrete [1]. The objective is to integrate environmental aspects into the product design in order to improve its environmental performance throughout its entire life cycle, and in particular its duration and residual treatment [2].

Concrete’s thermal behavior is relevant to any of its applications, especially in structural concrete where it is ideal to have low thermal conductivity, dimensional stability, high specific heat and little or no decrease in strength with increasing temperature.

Research is based on three pillars: use of concrete RAs; analysis of thermal conductivity, as the main parameter to measure energy efficiency; and the use of IRT as a qualitative, non-destructive technique for the study of thermal behavior. The importance of the study lies in the scarce research on thermal properties of this type of concrete, self-compacting with RAs, and validation of thermography as a first approach to the study of thermal conductivity, quantifying the results through a heat flow meter.

The current state of the research and publications in the three key areas of research is reviewed below.

### 1.1. Background of Recycled Aggregate

With the clear objective of prevention, the Directive 2008/98/EC establishes the legal framework for the treatment of one of the main current environmental problems such as the generation and management of waste from different industries, promoting reuse, and recycling. This Directive has been transposed to the different member states of the European Union, which in Spain was materialized in the current 2014–2020 State Program for Waste Reduction with the aim of reducing 10% of the waste generated in 2010 by 2020.

The main product of construction and demolition wastes recovery is the RA, granular material resulting from the treatment of inorganic materials previously used in the construction. Any facility that gives exclusive service to the construction work is considered an integral part of it, including precast concrete plants, among others. The need to reduce the final amount of waste produced in plants of precast concrete, with the intention of using it as RA in new parts manufacturing, thereby reducing the consumption of natural aggregates, is the target for energy efficiency this article is based on.

Construction and demolition wastes have been subject of study of many researchers in recent years. Behera *et al.* [3] have conducted a thorough review of the main results, ranging from the process of obtaining the RA; the analysis of the physical and chemical properties of the different types of RAs; analyzing the properties of recycled concrete both fresh and hardened state; and various techniques to improve the properties of RA, as well as the main issues on its use.

Out of the three RA types according to their origin (concrete, ceramic or mixed), recycled concrete aggregates object of this study may have two origins: traces of manufacturing (production disposed elements and tested specimens), or demolition of concrete structures. While in the first case the aggregates are relatively clean, with only cement paste attached; in the second case, the aggregate is often contaminated with salts, bricks and tiles, sand, dust, wood, plastic, cardboard, paper, and metals [4].

There are few countries that have regulations or recommendations of use for this type of aggregate for structural concrete, finding different trends in the required quality of RA, of the maximum allowed content in concrete, or limitation on its applications. Rilem [5], one of the first to be developed, and adopted in other countries such as Belgium or Hong Kong, pioneers in this field, allows the use of RAs with maximum absorption of 10% in concrete with category resistant to 50 N/mm^2^. The Japanese standard [6] is more stringent and fixes maximum absorption at 7% for concretes of category resistant up to 24 N/mm^2^, although the most demanding of all is the Spanish regulation EHE-08 [7], based on concrete made with coarse recycled aggregate from the crushing of concrete waste.

The required characteristics for RA’s concrete according to the EHE-08 that most affect this research are:

- Aggregates from concrete.
- Coarse aggregate with absorption <7%.
- Banned in precast concrete.
- Minimum aggregate size 4 mm.
- Replacement of natural aggregate by RA, maximum recommended 20%.
- Strength limit of 40 N/mm^2^.

This project focuses on the aggregate from crushing concrete pieces, which does not comply with quality control in a precast concrete plant in Galicia (Spain). It is reported that the recycled aggregates are relatively weaker than the natural ones, and therefore, compositional differences can be significant depending on the proportion of mortar present in the residue, which is the origin of the worst features of RAs, such as low density, low conductivity, and increased water absorption, Los Angeles coefficient of abrasion, and sulfate content. These characteristics also affect adversely the quality of the recycled concrete related elasticity, shrinkage and creep, as well as durability.

The expected properties of recycled concrete with RAs from concrete waste are [8,9,10,11,12]:

- Water demand in the fresh recycled concrete is higher than that of fresh concrete made by natural gravel and cement consumption for the same strength is somewhat higher.
- Recycled concrete density is lower than that of the original concrete; with 100% replacement of coarse aggregate, a density between 10% and 20% lower can be obtained.
- Substitutions of up to 30% of the conventional aggregate by RA do not significantly alter the compressive strength of the new concrete. When 100% of the coarse aggregate is replaced, the compressive strength may decrease between 10% and 20%.
- The elasticity module of recycled concrete is always lower (between 15% and 40%) than the reference concrete and reaches even smaller values when recycled fine aggregate is also used.
- The shrinkage and creep of recycled concrete are maintained when coarse aggregate replacement is less than 20%, whereas with a 100% replacement of coarse aggregate, shrinkage can increase up to 50% and creep between 30% and 60%. If fine recycled aggregate is also used, both values increase even more.
- For the same dosage, both absorption and porosity of recycled concrete increase. In reference concrete having 5%–6% absorption and 11%–13% porosity, absorption and porosity values between 8%–9% and 16%–20%, respectively, might be achieved.

The properties that may in principle be a problem, such as low density and high porosity, in this project are considered an advantage when designing elements aimed at, among others, serving as thermal insulator.

It would be worth mentioning, in view of highlighting possible concrete solutions with enhanced eco-friendly features, the possible role of fly ash in concrete with recycled aggregates, in terms of both mechanical performance [13] and thermal properties [14].

### 1.2. Background on Thermal Conductivity of Concrete

Different studies show that the three most influential factors in conductivity are the aggregate type, porosity and moisture.

- The factor that most affects is the type of aggregate used in its manufacture. If aggregate has higher conductivity, the concrete will also have higher conductivity. The thermal conductivity of natural aggregates is between 1.163 and 8.6 W/mK [15]. There are discrepancies regarding the temperature effect on the conductivity of natural aggregates. Some show a reduction in conductivity with increasing temperature, for example calcite, marble, limestone, dolomite; while others show little changes or slight increases with increasing temperature [16]. Such discrepancies lie in the reliability of the tests performed and the consideration or not of other factors that significantly influence the thermal conductivity, as the mineralogical composition of the aggregates themselves [17].
- The higher the humidity, the higher the conductivity [18]. This is because the conductivity of water is 25 times the conductivity of air; so when the air trapped in the pores is replaced by water, conductivity is increased. The relationship between the conductivity and moisture content is almost linear up to temperatures of 30 °C [17]. When the weight of the concrete increases by 1% due to water absorption, the thermal conductivity increases by 5% [19].
- The thermal conductivity of concrete increases with cement content, as with higher amounts of fine gaps between the grains are much smaller [18].
- The thermal conductivity is related to the density, higher conductivity equals higher density. According to Uysal [19], for a specific concrete mixture, when the amount of cement increases by 25%, the density increases and therefore conductivity increases by 3%.

### 1.3. Background of Infrared Thermography (IRT)

IRT is widely used as a non-destructive test to evaluate the behavior of a material without destroying it or interfering in its lifespan. It can be used in different applications (military, industrial, civil engineering and medicine) in which the variation of the surface temperature may indicate a problem or characteristic of the material [20].

In particular, in construction field, it has been used since the 1990s in many fields demonstrating its suitability in [21,22,23] assessing the insulation of facades; localization of heat losses (building envelope, thermal bridges, doors and windows); detecting moisture in building structures; and inspecting and maintaining electrical and mechanical building installations. More specifically, in concrete, both as a qualitative method and method combined with numerical simulation to quantify analysis to [24,25,26,27,28], IRT can detect cracks or defects; evaluate structures; locate reinforcement in the concrete; detect gaps or voids; and can even be used as a technique for comparative analysis of different materials. In some cases, electrical impulses, microwave, or heat sources are used to analyze the thermal behavior of materials.

According to Clark *et al.* [29], the main advantages of IRT are needless direct contact between the camera and the object of study; thermal cameras capable of analyzing the temperature in several different places at once; greater visibility in respect of thermal radiation that is affected by smoke or mist; measurements in wide temperature range from –20 to 1600 °C; equipment capable of detecting temperature fluctuations precisely within ±0.08 °C; lightweight and portable thermography equipment; and finally, the recorded data can be monitored and processed in a standard computer with a specific imaging software. In contrast, the major disadvantages are radiation reaching the thermography system depends not only on the temperature, but also on the emissivity of the material; a material with emissivity lower than 1 reflects radiation from surrounding objects and may alter the registered temperature; and radiation is attenuated in the atmosphere due to absorption of energy by the particles in suspension and this generates random re-radiation that may produce the results’ alteration (these effects can be considered negligible when the distance between the object and the camera is small).

Despite the aforementioned disadvantages, which might create some apprehension regarding the suitability of this technique, it is worth noting that these problems are due in large part to lack of experience, misapplication or misinterpretation. That is why this research aims to enhance the uses of IRT, seeking a pioneer application as qualitative analysis of the thermal conductivity.

## 2. Experimental Program

The stages in which the experimentation is developed range from obtaining RA, through the manufacturing and preparation of recycled self-compacting concrete (RSCC) specimens, to the completion of mechanical and thermal tests.

### 2.1. Recycled Aggregate Production

The pieces rejected throughout its manufacturing process undergo a thorough control that guaranties greater homogeneity of RA in comparison to products manufactured by concrete from demolition, ensuring that there is no contamination with external agents.

The origin products that have served as raw material for the production of RA are made from the use of two types of concrete products: dry concrete and self-compacting fluid concrete. The prestressed slabs and hollow core slabs are made with dry concrete, while the walls, columns, and beams are made with prestressed SCC. Materials used were: cement CEM I-52.5 R; fine aggregate, with an aggregate size less than 2.5 mm (0/2.5); coarse aggregate, with an aggregate size less than 5 mm (0/5); gravel aggregate, with an aggregate size from 6 to 12 mm (6/12); calcareous filler-A; high range water reducer additive (V-20 HE); and water. Table 1 shows the dosages of each product.

**Table 1:** RA origin dosages.

Original Product	Cement CEM I [kg/m^3^]	Sand 0/2.5 (quartz) [kg/m^3^]	Sand 0/5 (quartz) [kg/m^3^]	Gravel 6/12 (granite) [kg/m^3^]	Additive V-20 HE [L/m^3^]	Filler A calcareous [kg/m^3^]	Water [L/m^3^]
P. slab, SCC-45	303	680	450	770	7	-	107
H. slab, SCC-45	350	400	475	1050	1.5	-	133
Wall, SCC-45	275	-	950	800	4.7	300	124
Column, SCC-55	315	-	925	860	5.5	290	142
Beam, SCC-60	350	-	890	810	5.5	280	158

Obtaining RA is done at the factory. The process consists of pre-shredding all parts with a hammer crusher leaving the material in an approximate size of 300 mm. Then, the reinforcement is separated from the concrete. Later, by jaw crusher, the aggregate size is reduced to a size between 40 and 80 mm, and the smaller scrap metal is removed on a conveyor belt. With the aggregate free from steels, a new crushing process with a crushing mill and subsequent screening is performed by a band to select an aggregate size less than 12 mm (0/12 fraction). As a final stage, a wash process is performed.

The composition of the RAs obtained is 40% fine (sand 0/5) and 60% coarse (gravel 6/12). This is therefore a mixture of fine and coarse aggregates. Although the EHE-08 only recommends the use of recycled coarse aggregate, it has been decided to focus research on compositions for two reasons: the first one is that the aggregate processing method is so obtained and the other one is to check that, although nowadays it is not provided by law, from experience it is known that there are more options that will end up being included in the legislation, widening the spectrum of aggregates utilization.

### 2.2. Design and Preparation of Specimens

SCC is able to flow and fill anywhere in the formwork through the reinforcement simply by the action of its own weight without using any method of vibration or compaction. Such benefits are achieved with fluidity, viscosity and appropriate cohesion in the mixtures of these concretes. The key to obtain a highly cohesive SCC with good self-compacting properties lies in the correct optimization of the composition of the cement paste, especially the combination of water, superplasticizer and viscosity modifying agent. This latter will negatively affect the fluidity, so to obtain a specific workability the amount of water and/or dosage of superplasticizer must be increased [30,31].

For RSCC-50 manufacture, the dosages used in the plant for the product manufacturing process without RAs remain constant, taking the substitution of the aggregate fractions by RA of self-consumption as a variable, with the following substitution percentages: 0% (reference SCC), 20% (the maximum substitution recommended by EHE-08), 50% and 100%.

The selected reference mixture consists of the following materials: cement CEM I-52.5 R; calcareous filler-A; fine quartz aggregate size 0/5 and 0/2.5; granite coarse aggregate size 6/12; Viscocrete 20 HE additive; and water.

Early trends in the design of concrete mixtures with RAs led to increased cement content to reduce the loss of compressive strength generated by the same aggregate [32]. However, increasing the cement content generates significant inconveniences in environmental and durability terms. In this research project, the intention is to make use of the content of the RA mortar to minimize the cement content, and thus obtain a conventional equivalent resistance without compromising durability aspects.

In Table 2, the dosages are taken to each proposed substitution, 0%, 20%, 50% and 100%. The RA has not been pre-wetted before use. The amounts of cement, filler and additive have remained constant, and only the proportions of aggregate have been modified, except for sand 0/2.5, which has remained virtually constant in percentage. The theoretical water/cement (w/c) ratio is 0.45; however, the final ratio is shown in the table, since producing concrete in an automatic concrete mixer, this corrects the amount of water depending on the workability of each kneaded, measuring the moisture with an ammeter. It needs to be considered that with larger amounts of water, a lower initial resistance might be present, lower resistance evolution, higher possibility of quality defects such as shrinkage, carbonation, evaporation, *etc.* With these dosages, 20 specimens of 25 × 25 × 5 cm^3^, are done: five per dosage type. The dimensions have been chosen in such a way to evaluate different properties both as infrared thermography and for the study of the thermal conductivity.

**Table 2:** Recycled self-compacting concrete RSCC-50 dosages.

Type	Cement [kg]	Filler [kg]	Additive superplasticizer (V–20 HE) [L]	Fine Sand 0/5 (quartz) [kg]	Superfine Sand 0/2,5 (quartz) [kg]	Gravel 6/12 (granite) [kg]	RA 0/12 [kg]	Water [L]	w/c
A	335	320	5.4	0	0	0	1,6400	180	0.54
B	335	320	5.4	48	367	391	817	166	0.50
C	335	320	5.4	335	367	604	327	154	0.46
D	335	320	5.4	540	390	760	0	151	0.45

### 2.3. Mechanical Tests

The purpose of these tests is not making a comprehensive analysis of the mechanical properties of concrete obtained, but to merely confirm the suitability of the RAs of consumption as feedstock, hence it was decided to perform the tests to the reference specimen and to that of the 20% recommended in the EHE-08, further testing to the other dosages being ruled out. The study of concrete with percentages of substitution higher than 20% is focused on the search of minimum operating workability mixtures, as well as its thermal analysis.

The main parameters that characterize a SCC in both states, fresh and hardened, were tested:

▪ Fresh: consistency control by slump-flow testing according to UNE-EN 12350-8: 2011 [33].
▪ Hardened state: determination of compressive strength according to UNE-EN 12390-2: 2009 [34]. For this purpose, eight standard cubic samples of 10 × 10 × 10 cm^3^ were made, four of the mixtures without RA (S1–S4), and the other four of the mixtures with 20% of RA (S5–S8), which were tested within three and 28 days. Seven cylindrical specimens of 15 × 30 cm^2^ were also prepared with the 20% mixture, tested within 28 days (S9–S15). Curing of the specimens was performed in a moist chamber until the date of rupture.

### 2.4. Thermal Tests Using Infrared Thermography

#### 2.4.1. Design of Testing Cabin

A cubic cabin 1.20 m each side is constructed, according to Cerdeira *et al.* [35]. One of the walls is a typical facade cladding, consisting of: double hollow brick, 1.5 cm thick mortar and gripping mortar. To give more strength to the set, small sidewalls, which give stability to overturning, are built.

**Figure 1:**
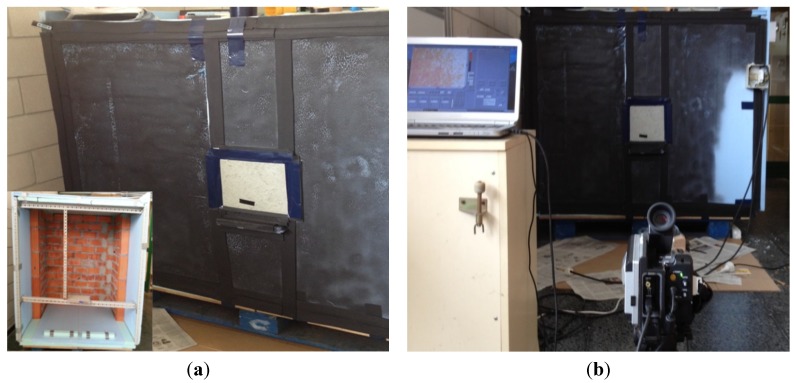
(**a**) Cabin painted black with specimen centered. Bottom left: cabin without the front panel, with support area for the specimen; (**b**) Measuring and recording equipment.

A metallic profiles structure bears on the other three walls, rigid and opaque with a double layer of insulation, extruded polystyrene, positioned staggered, so that at any position matching tongue and groove joints of the panels are different. This structure serves as a support for reinforcing the test specimen standing in the removable wall. The front wall is painted black to prevent reflections and improve the quality of thermal images (Figure 1a).

#### 2.4.2. Measuring Systems

Once the cabin is built, a control unit is designed; such unit being capable of evaluating various temperature sensors placed, four of them on the inner walls, and two more for room temperatures inside and outside the cabin. To keep the inner thermal gradient, a 2 kW electric air heater placed inside is used, and controlled by the unit configured by programming its function blocks. 

This unit is responsible for calculating, each time, the temperature difference between the two sensors, controlling the relay activation or deactivation, which allows switching on or off the heater unit to maintain constant temperature inside the cabin. All temperatures recorded are sent to the computer via the communications adapter. Finally, to protect the equipment and circuits against short circuit and overload, a magnetothermic breaker is used. In Figure 1b, the installation assembly with the thermal camera is observed.

#### 2.4.3. Infrared Radiation Equipment

As it is known, infrared cameras transform thermal energy radiated by objects in the infrared range of the electromagnetic spectrum in a visible image where each energy level is represented by a color or gray scale. Objects emit energy proportional with their surface temperature according to Planck’s Law, and that energy is converted to temperature by the Stefan-Boltzmann Law. The energy detected by the camera really depends on the emissivity of the surface to be measured and on environment. In fact, the atmosphere between the object and the camera absorbs some of this energy and other energy is added when reflected from the surrounding surfaces. 

The technology used in this research, for forming images, is focal plane with multiple-detector of elements in a matrix, on which the object surface radiation is focused, all points simultaneously conforming image (or pixels). There is a direct relation between detecting element size and the capacity thereof to capture radiation: as the detector size is made smaller (increasing the number of pixels forming the image), the spatial resolution of the image becomes greater, and thermal resolution decreases due to less sensitive surface of the measuring elements. Therefore, a compromise between optical and thermal resolution is necessary.

The camera has a detector with a matrix of 640 × 480 microbolometer integrated with a digital color camera of 1280 × 1024 pixels and can superimpose the two images. It has a measurement range of −40 to 500 °C, a visual field of 21.7 °C (H) × 16.4 °C (V), accuracy of ±2% of the reading value, a resolution of 0.06 °C at 30 °C, and a spectral range of 8 to 14 µm. The camera, thermally calibrated against a black body simulator at an ISO certified laboratory, is also geometrically calibrated according to the principles of photogrammetry using a field calibration based on emissivity, prior to testing.

The monitoring of the thermal behavior of 20 specimens with four different RA compositions, at 20 °C, is done creating a quasi-steady temperature difference between the interior, with constant temperature of 50 °C, and as a result, an exterior temperature of 30 °C. The maximum temperature reached by each face of the specimen and the time required to achieve it is analyzed. Through the thermal camera by appropriate software, images are recorded every two minutes, which allows observing the transient heating of each specimen. The average duration of tests is 165 min.

**Figure 2:**
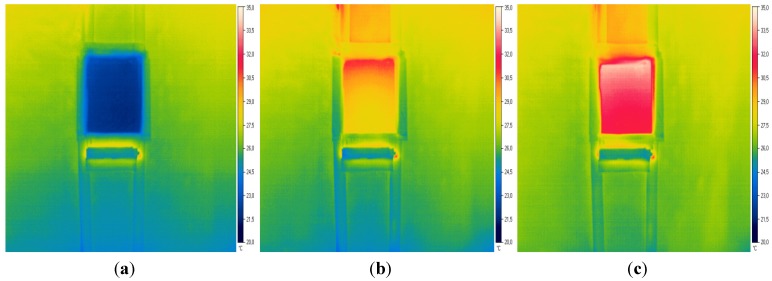
(**a**) A1 specimen thermography at t = 0, with T = 21.82 °C; (**b**) A1 specimen thermography at t = 90 min; (**c**) A1 specimen final thermography at the end with T = 32.6 °C.

Prior to conducting the thermography to the specimens, thermal emissivity is measured by an isolating tape in accordance with the Fokaides *et al.* test [36], getting a value of 0.94–0.96. The cabinet average warming period, before starting measuring, is 150 min. Figure 2 shows the thermal images of A1 specimen at three points of inspection: first, with a temperature of 20 °C; 90 min later, with an average surface temperature of 30 °C; at the end of the test, 170 min later, where the specimen reaches a temperature of 32.6 °C.

### 2.5. Thermal Tests with Heat Flow Meter

#### 2.5.1. Measuring Equipment

To determine thermal conductivity in steady state of the 20 RSCC specimens at three temperatures, 20 °C, 25 °C and 30 °C, a symmetric heat flow meter from a single sample in portrait orientation is used, according to UNE-EN 12667:2002 [37], applicable to all products with an expected thermal resistance equal to or greater than 0.5 m^2^K/W.

The equipment, a commercial LM.305 with own control software and acquisition data, is equipped with two plates, one fixed and one mobile. In the central area of each plate, the measurement zone, one heat flow meter is embedded provided with thousands of small thermocouples. The average signal of each heat flow meter is proportional to the heat flow through the specimen and that signal is used to determine the thermal conductivity of the specimen.

The test consists of applying a temperature gradient, programming plates at different temperatures to produce a heat flow through the specimen. The temperature difference is calculated as the difference between the temperatures measured in the center of each plate. To accurately determine the thermal conductivity of the material, it must be at steady state, *i.e.* it must meet a number of criteria as:

▪ Temperature criteria: the plate’s temperature must be stabilized. The technical specifications of the equipment manufacturer LM.305 specify temperature fluctuation below ±0.01 °C.
▪ Criteria at the output signal for the heat flow meters: the signal of the heat meters should not vary. According to the manufacturer’s specifications, heat flow is measured with two separate sensors with a precision access of 0.01 W/m^2^.

In this manner it is possible to determine the material thermal conductivity at steady state by using the following equation derived from Fourier’s Law:

(1) $$\frac{\text{dQ}}{\text{ΔT}}\;=\;\frac{\text{λ}}{\text{d}}\text{​ }=\;\frac{1}{\text{R}}$$

where dQ is the heat flow, obtained from the calibration constants of the heat flux meters and the electrical signal provided [W/m^2^]; ΔT, the temperature difference established between the two sides of the specimen [K]; λ, the specimen thermal conductivity [W/mK]; d, the specimen thickness [m]; and R, the thermal resistance of the specimen [m^2^K/W].

#### 2.5.2. Specimens Preparation and Measurement

The 20 specimens conditioning to meet the standard specifications is first a polishing process to make the top face flat; the other faces are already parallel plates because of the characteristics of the mold in which they were made; a dimensional control with a caliber which permits the remaining specifications. A series of measurements are performed (three length and width, and three on each side of the specimen A, B, C and D) and weighed on milligram scale. Density is calculated from the dimensions of the specimen, the thickness measured and the mass of the specimen.

**Figure 3:**
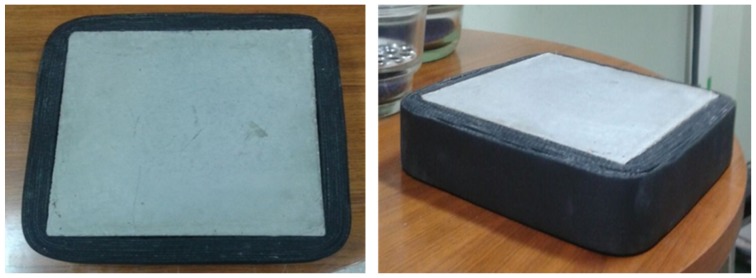
Specimens awaiting preparation and verification.

Furthermore, the surface of the heating units is 30 × 30 cm^2^ and the dimensions of the specimens are 25 × 25 × 5 cm^3^. To ensure the steady state, units are surrounded with insulation thereby avoiding heat losses at the edges. The final dimensions of the specimens with the tape are 30 × 30 × 5 cm^3^ (Figure 3).

**Figure 4:**
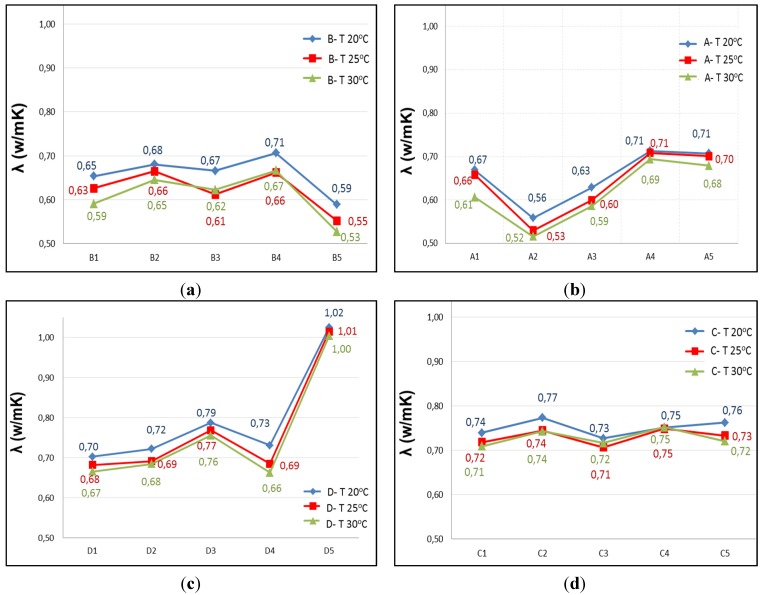
Conductivity at 20 °C, 25 °C and 30 °C: (**a**) Type A with 100% RA; (**b**) Type B with 50% RA; (**c**) Type C with 20% RA; and (**d**) Type D with 0% RA.

Given the characteristics of the equipment and the graph shown on the computer screen representing the variation of heat in time, the measurement time is estimated as the slope of the curve j = f(t) is constant. Tests are performed at three temperatures: 20 °C, 25 °C, 30 °C. In Figure 4, the results of the five specimens of each type Are reported. 

There is some variability in the data, yet they show the same behavior at the three temperatures, so it is discarded as being caused by faults arising in the thermal tests. It is attributed to the composition of each specimen with influence in the homogeneity of the mixture. The RAs have 40% of fine and 60% coarse, so it seems that there may be specimens with higher percentage of coarse aggregate than others, hence the composition of the mixture is less uniform, with more voids or porosity, thereby affecting the conductivity. This effect may be more pronounced in the specimen with the highest percentage of substitution, as A type.

In addition to variability, there are a number of different values on others of its type or “outliers”: the three data from A2, the data from B5, and especially the three data from D5. With the intention of detecting whether or not values are atypical, the Tukey test is performed on all the specimens of Groups A, B and D. The result is that neither A nor B have outliers, however values on specimen D5 are slight outliers. This test also shows that C type is showing less scattering in the data, smaller boxes, and more focused mean.

At this point, in view of the results obtained in the different analysis and considering that the specimens D correspond to the reference sample containing no RA, it was decided not to consider the specimen D5, excluding it from the investigation. The results indicate that there must have been a failure in the preparation of the mixture, a fact that was also evident with a weight 2% higher than the average weight of type D and a higher density, so it can be deduced that it had a content of fine higher than it should for that mixture. From here the mean data of the specimens D are referred only to the remaining four samples: D1, D2, D3 and D4 and are displayed in the results section of this article.

## 3. Results and Discussion

### 3.1. Results and Discussion of the Basic Mechanical Properties

#### 3.1.1. Fresh SCC

Consistency control through the slump-flow test gave patty diameters of 720 mm for the reference mix (0%) and 670 mm for that of 20%. Both comply with the legislation under which the diameter should be between 550 and 850 mm [7,38]. It may be observed that no segregation happens since there is no mortar exudation on the peripheral zone and that the aggregate concentration is uniform.

#### 3.1.2. Hardened SCC

Table 3 shows the results of compressive strength for the different specimens. The average values of compressive strength are: for the specimen with 0% RA, after three days 48.75 N/mm^2^ and after 28 days 62.25 N/mm^2^; and for the sample having 20% RA, after three days 46.35 N/mm^2^ and after 28 days 54.6 N/mm^2^.

These values are obtained by applying the appropriate equivalency conversion factor from cubic to cylindrical specimens in order to be able to objectively compare the results. Furthermore, the average compressive strength of the seven cylindrical specimens having 20% RA after 28 days is 54.41 N/mm^2^.

**Table 3:** Compressive strength results (N/mm^2^).

Cubic Specimen	3 days	Cubic Specimen	28 days	Cylindrical Specimen	28 days
S1 (0% RA)	49.3	S3 (0% RA)	60.3	S9 (20% RA)	56
S2 (0% RA)	48.2	S4 (0% RA)	64.2	S10 (20% RA)	50.3
S5 (20% RA)	47.3	S7 (20% RA)	54.8	S11 (20% RA)	54.9
S5 (20% RA)	45.4	S8 (20% RA)	54.4	S12 (20% RA)	52.6
				S13 (20% RA)	57.5
				S14 (20% RA)	53.3
				S15 (20% RA)	56.3

In view of the results shown in the mechanical tests, the following conclusions can be drawn:

▪ Starting from the basis that the percentages of cement and filler have remained constant, it is found that water demand is greater when the percentage of RA increases. Specifically compared with the reference specimen without RA, it increases by 2% when replacement is 20%; by 10% when the replacement is 50%; and by 19% when RA replaces the entire aggregate. Since the amount of cement is constant, this water increase directly affects the w/c ratio, which starts at 0.45, and becomes 0.55 for the mixture with 100% RA.
▪ The values obtained from the compressive strength confirm that the resulting recycled self-compacting concrete (RSCC) has a resistance greater than 50 N/mm^2^. Knowing the possible decrease in resistance when using RAs, because of previous research [8,11,19], a mixture with more characteristic resistance to meet this requirement has already been selected, exceeding the recommendations of EHE-08.
▪ From the tests performed, it is concluded that the average compressive strength of the cubic specimens decreases by 5% after three days when RA replaces 20%. Under the same conditions, but after 28 days, the average compressive strength is reduced by 14%.
▪ In slump-flow tests, by the difference in patty diameters, a difference of 7% workability is seen. The mixture with 20% RA, despite having more water, shows less workability than the one with 0% RA because of higher irregular fines coming from RAs. Remember that the composition of RAs is 40% of fine aggregate and 60% coarse aggregate.
▪ Tests in hardened state show that the evolution curve for resistance is smoother in concrete with RA than the one on the reference mix (smaller slope). Nevertheless, the reference concrete reaches on the third day 78% of its strength within 28 days, whereas concrete with 20% RA on the third day reaches 85% of its strength within 28 days.

It is therefore recommended that in early installations of these RA concretes, one requires that compressive strength after three days is at least 90% of the required.

### 3.2. Results and Discussion of the Thermal Properties

#### 3.2.1. Assessment for the Thermal Behavior Using Thermography

The duration of the test is 90 min. In Table 4 the results of the thermographies of the following parameters are shown: specimen type based on the percentage of RA (A, 100%; B, 20%; C, 50%; and D, 0%); final average surface temperature of each specimen, T_fin_ (°C); average temperature increase of the evolution of the temperature of the outer face of the specimen, ΔT; average gradient heating, α (°C/min); average test duration, t (min). In addition, a variable, τ, is introduced as the time it takes to reach 99% of the final temperature.

**Table 4:** Thermography results for each specimen type.

Type	% RA	T_fin_ (°C)	ΔT	α (°C/min)	t (min)	τ (99% T_fin_)
A	100	28.42	7.04	0.044	154.4	162.8
B	50	28.76	7.64	0.048	159.6	174.8
C	20	29.68	9.77	0.056	176	163.2
D	0	30.77	9.84	0.056	169.5	159.5

**Figure 5:**
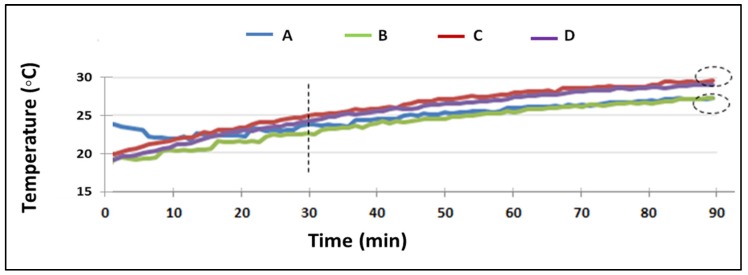
Evolution of the average temperatures of each specimen type.

To better analyze the results, Figure 5 shows the evolution of the average temperature of each type throughout the test. The existence of two distinct groups, according to their ability of heat transfer (black-dotted ellipses), shows perfectly, as a first conclusion: on the one hand, Groups A and B, with similar ΔT, α and t, have a lower final temperature and thus more thermal resistance to heat flow. Furthermore, Groups C and D, with similar ΔT, t and α, will take less effort to increase the temperature. The maximum difference in T_fin_ is 2.35 °C, between type A (100% RA) and D (0% RA). The separation of groups becomes visible from half an hour of the assay (dashed line). Analyzing the τ variable, it can be concluded that the thermal inertia of the specimens are remarkably different. There are 15 min of difference between the type D specimens that takes less time to reach 99% of its final temperature and B type wich are the ones that take longer. In between, the types A and C have a similar thermal inertia.

#### 3.2.2. Evaluation of Thermal Conductivity by the Heat Flow Meter

Table 5 shows the weights, densities and average conductivities for each specimen type at all three temperatures. Note that for Groups A, B and C, it is the average of the five specimens of each type, but for Group D there are only four specimens. In the experimental section of this article it is justified why D5 specimen was discarded.

**Table 5:** Average conductivities at the three temperatures.

Type	% RA	Weight kg	Density kg/m^3^	Average λ (W/mK)		
				T (20 °C)	T (25 °C)	T (30 °C)
A	100	7.11	2,129.97	0.655	0.639	0.616
B	50	7.26	2,211.97	0.659	0.623	0.610
C	20	7.32	2,292.50	0.751	0.730	0.728
D	0	7.60	2,339.97	0.736	0.707	0.692

Regarding the weight, a decrease is seen when increasing the RA content; however, a trend may not be set with sufficient correlation to be representative. The materials from which the recycled aggregates proceed are very similar to the components of the reference mixture, and the weight reduction is mainly due to the increase of voids in the concrete by the irregular shape of the recycled aggregate.

**Figure 6:**
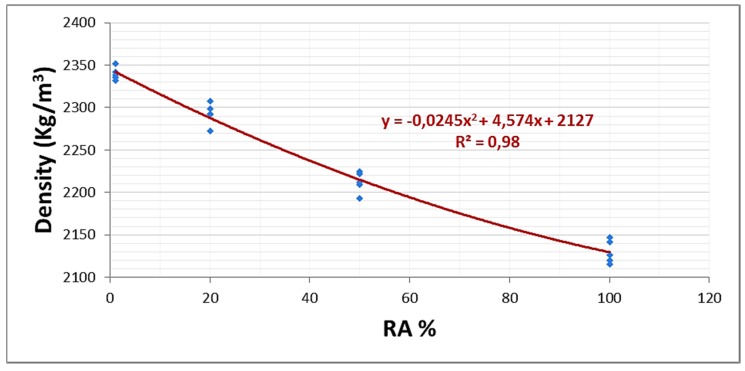
Relation between RA % and density.

Analyzing densities, a polynomial relation is obtained (Figure 6), in which the density lowers when the RA content is increased. Specifically, for 100% RA, there is a 9% reduction, which is a limit lower than indicated in the introduction to this article.

**Figure 7:**
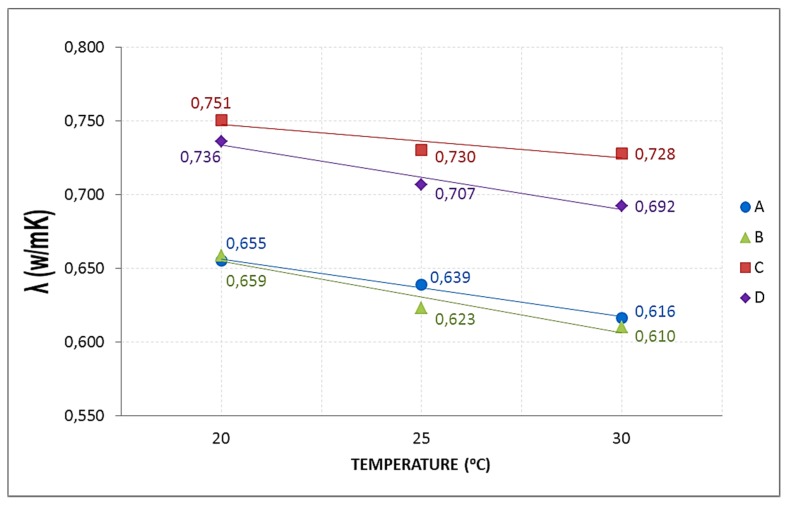
Influence of temperature on the thermal conductivity.

When studying the effect of temperature on the average conductivities, it might be observed that the trend is a linear decrease, *i.e.*, average conductivity decreases when the temperature is increased (Figure 7). All specimens have the same relation, same slope, except for C, whose slope is smaller. For D, B and A, each 5 °C, the average temperature is increased, it means that thermal conductivity decreases by 3.25%. With Group C, the relation is half, which means, per every 5 °C the average temperature is increased, the conductivity decreases by 1.6%. The slope in the line equation is the reason.

**Figure 8:**
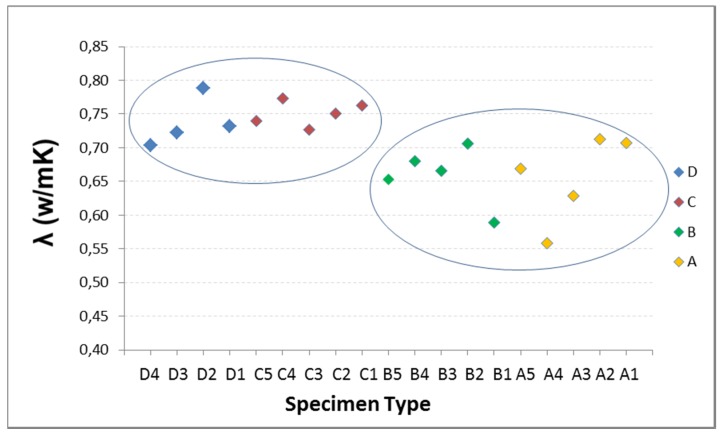
Influence of recycled aggregate percentage on thermal conductivity at 20 °C.

**Figure 9:**
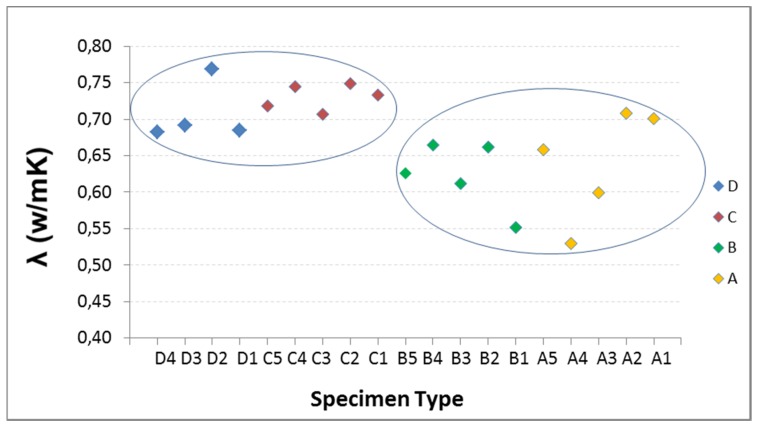
Influence of recycled aggregate percentage on thermal conductivity at 25 °C.

**Figure 10:**
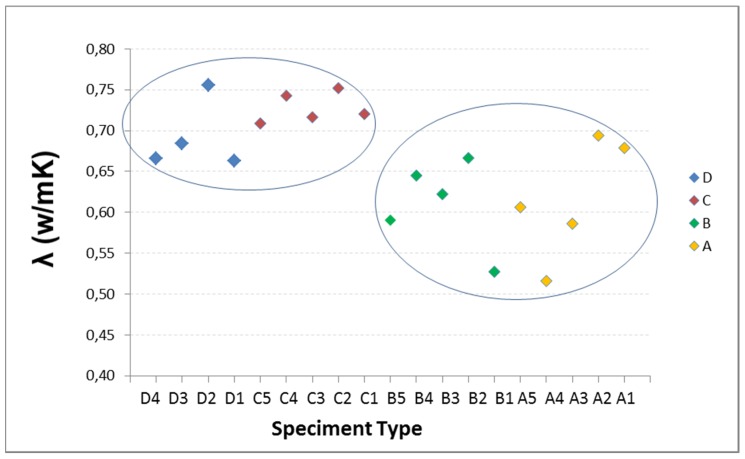
Influence of recycled aggregate percentage on thermal conductivity at 30 °C.

Below, the influence of RA percentage on thermal conductivity is studied at three temperatures: 20 °C, 25 °C and 30 °C (Figure 8, Figure 9 and Figure 10).

The thermal conductivity and the RA content do not show a clear trend. Therefore, it may be stated that there are two very different thermal behaviors represented in Figure 8, Figure 9 and Figure 10 by the two ellipses: the specimens with lower percentage of RA and higher conductivity (D and C), and secondly those containing higher percentages of RA and lower conductivity (B and A).

From the different studies conducted and the literature reviewed, it can be said that the general trend is: when the percentage of recycled aggregate increase, the thermal conductivity decrease. However, besides the RA content, other factors influence on thermal conductivity, such as type and form of aggregate, temperature, mixture composition, *etc.* These are the factors that explain the differences in conductivity values.

In the D and C groups, the average conductivity of C is slightly higher than of the D type. It may be, on the one hand, because the D5 specimen has been ruled out as an atypical value (Tukey test), the one with the highest conductivity, which makes that the average conductivity considerably reduced; in C, the conductivity is the average of the five samples. On the other hand, the composition of the mixtures is different: the C specimens have a higher content of natural super-fine aggregate size 0/2.5 mm, 44% of the total aggregate compared with 40% for D; a factor that can influence on conductivity, increasing slightly the conductivity of C with respect to the D.

By increasing temperature, conductivity decreases; and the D samples are more sensitive to this factor than to RA content. However, this increase of 0.04 at 30 °C, in the worst case, is not considered representative since the range of variation in conductivity measurements, for this group, is 0.07. Hence it may be stated that for recycled aggregate percentages between 0% and 20%, recycled aggregate is not a determining parameter in the thermal conductivity.

In the B and A groups, the RA contents are higher than in D and C. The recycled aggregates used have a composition of 40% with size 0/6 mm and 60% with 6/12 mm. This aggregate is mixed, not separated in the two sizes, and for this reason, when the aggregate is selected for adding in the mix design, in each mixture may be samples with greater fine aggregate content than others, and therefore the void fraction and the pore size in concrete is lower, increasing the conductivity. In addition, the recycled aggregates are more irregular and come from materials with different conductivities, thus causing greater variability of the conductivity data with increasing the RA content, as is shown in Figure 8, Figure 9 and Figure 10.

In these groups, at room temperature (20 °C), average conductivities of A and B are equal. As temperature increases, conductivity is lower; and the B specimens are more sensitive to this factor that the RA content. Hence it may be stated that for recycled aggregate percentages between 50% and 100%, recycled aggregate is not a determining parameter in the thermal conductivity.

The final stage is that the RA content influences on conductivity for AR percentages between 20% and 50%. The reduction of the average conductivity of Group C (20% of RA) compared to Group B (50%) is 15%.

## 4. Closing Remarks

The experimental study of specimens RSCC-50 with different dosages of RAs (0%, 20%, 50% and 100% substitution of fine and coarse aggregate by RA) has drawn the following conclusions:

1- The specimen having 20% RA, compared to the reference, has 7% less workability; an increase of 2% in water demand gives a 0.46 w/c ratio; a decrease in compressive strength after three days by 5% and by 14% after 28 days; smoother evolution curve reaching on the third day 78% of the strength at 28 days. Based on this last assumption, it was proposed as a recommendation of use that, for early RSCC facilities with 20% RA, 90% of the required strength is reached within 3 days. These results corroborate, justify and quantify existing research outlined in the theoretical framework of this research.
2- The weights of the specimens decreased with increasing % of RA with no clear trend. However, the decrease in density with increasing % of RA follows a polynomial relationship.
3- The higher temperature and higher % of RA make that data variability in thermal conductivity higher. C type specimens (20%) are those whose data have lower dispersion, so they are more stable and have better reproducibility.
4- With increasing temperature, the conductivity decreases linearly. In D, B and A specimens per every 5 °C increase in temperature, conductivity decreases by 3.25%. In C specimens. however, this relation is half, being less susceptible to temperature variations.
5- The general trend is that increasing the RA percentage the conductivity decreases significantly, mainly from a percentage of 20% in forward in which reductions of 17% are reached at 30 °C. However, this inverse negative relation does not exhibit a strong enough correlation.
6- Nevertheless, what can be established about the influence of RA percentage on the conductivity is that there are two distinct thermal behaviors appreciated both qualitatively (IRT) and quantitatively (heat flow meter): the A and B type specimens have lower and constant conductivities for each temperature, whereas C and D type specimens have 15% higher conductivities, although they do not have equal conductivities if they are in the same range.
7- The main differences between the samples of the second group are the average thermal conductivity, which in C type specimen is slightly higher than that of D type, 2%; reduction in conductivity with increasing temperature is lower in C than in D, by half. Despite the mechanical and thermal differences set out between C and D, specimen C, it may be concluded, corroborating the provisions of EHE-08, that the final properties of concrete are slightly affected in relation to those presented by conventional concrete.

## 5. Conclusions

In order to improve the sustainable aspect of self-compacting concrete incorporating recycled aggregates in manufacturing to enhance their thermal properties, an experimental study has been carried out.

After confirming that the recycled self-compacting concrete is feasible for structural applications, the thermal analysis suggests an appreciable improvement in thermal behavior in comparison with the reference mix without AR.

As an overall conclusion, it can be said that the thermal conductivity is reduced by 15% to increase the AR% from 20% to 50%. This is why the study of the replacement percentage of aggregates with RA comprised between 20% and 50%, with the intention of determining the percentage of RA at which thermal conductivity stabilizes, is proposed as a future research.

Following IRT analysis, its suitability as sampling technique for estimation or deduction of the thermal behavior in materials with a very small range of variation in conductivity, resistance and thermal inertia is confirmed.
